# Myopia and axial length in school-aged children before, during, and after the COVID-19 lockdown–A population-based study

**DOI:** 10.3389/fpubh.2022.992784

**Published:** 2022-12-15

**Authors:** Wei Pan, Jiang Lin, Li Zheng, Weizhong Lan, Guishuang Ying, Zhikuan Yang, Xiaoning Li

**Affiliations:** ^1^Aier Institute of Optometry and Vision Science, Aier Eye Hospital Group, Changsha, China; ^2^Chengdu Aier Eye Hospital, Chengdu, China; ^3^Education Bureau of Qingyang District, Chengdu, China; ^4^Aier School of Ophthalmology, Central South University, Changsha, China; ^5^Aier School of Optometry and Vision Science, Hubei University of Science and Technology, Xianning, China; ^6^Guangzhou Aier Eye Hospital, Jinan University, Guangzhou, China; ^7^Center for Preventive Ophthalmology and Biostatistics, Department of Ophthalmology, Perelman School of Medicine, University of Pennsylvania, Philadelphia, PA, United States; ^8^Changsha Aier Eye Hospital, Changsha, China; ^9^Hunan Province Optometry Engineering and Technology Research Center, Changsha, China; ^10^Hunan Province International Cooperation Base for Optometry Science and Technology, Changsha, China

**Keywords:** myopia, COVID-19, prevalence, axial length (AL), Chinese young students

## Abstract

**Background:**

Myopic shift had been observed during the COVID-19 lockdown in young school children. It remains unknown whether myopic shift is accompanied with increase in axial length. We aimed to evaluate the impact of the COVID-19 lockdown on myopia and axial length of school children in China by comparing them before, during and after the lockdown.

**Methods:**

In this population-based cross-sectional study, school-based myopia screenings were conducted in the Fall of 2019, 2020, and 2021 (representing before, during and after COVID-19 lockdown respectively) in Chengdu, China. Myopia screenings were performed on 83,132 students aged 6 to 12 years. Non-cycloplegic refractive error was examined using NIDEK auto-refractor (ARK-510A; NIDEK Corp., Tokyo, Japan) and axial length was measured using AL-Scan (NIDEK Corp., Tokyo, Japan). Spherical equivalent (SER, calculated as sphere+ 0.5^*^cylinder), prevalence of myopia (SER ≤ -0.50 D), and axial length were compared across 3 years stratified by age.

**Results:**

Myopia prevalence rate was 45.0% (95% CI: 44.6–45.5%) in 2019, 48.7% (95% CI: 48.3–49.1%) in 2020, and 47.5% (95% CI: 47.1–47.9%) in 2021 (*p* < 0.001). The mean non-cycloplegic SER (SD) was −0.70 (1.39) D, −0.78 (1.44) D, and −0.78 (1.47) D respectively (*p* < 0.001). The mean (SD) axial length was 23.41 (1.01) mm, 23.45 (1.03) mm, and 23.46 (1.03) mm across 3 years respectively (*p* < 0.001). From the multivariable models, the risk ratio (RR) of myopia was 1.07 (95% CI: 1.06–1.08) times, the SER was 0.05 D (95% CI: 0.04 D to 0.06 D) more myopic and the mean axial length increased by 0.01 mm (95% CI: 0.01 mm to 0.02 mm) in 2020 compared to 2019. In 2021, the risk ratio (RR) of myopia was 1.05 (95% CI: 1.04–1.06), the mean SER was 0.06 D (95% CI: 0.05 D to 0.07 D) more myopic, and the mean axial length increased by 0.03 mm (95% CI: 0.02 mm to 0.04 mm) compared to 2019.

**Conclusions:**

The COVID-19 lockdown had significant impact on myopia development and axial length, and these impacts remained 1 year after the lockdown. Further longitudinal studies following-up with these students are needed to help understand the long-term effects of COVID-19 lockdown on myopia.

## Key messages

- COVID-19 postpone great burden on children's myopia development.
- Myopia risks brought by the COVID-19 lockdown is accompanied with axial length elongation.
- Even as social restriction policies loosen in 2021, the effect of COVID-19 lockdown on myopia still remained.

## Introduction

Myopia, a major cause of irreversible blindness, is a major public health concern worldwide (1). According to the World Health Organization's projection made in 2016, about 50% of all population will be myopic by the year 2050 (2). The nationwide school closure from the outbreak of coronavirus disease 2019 (COVID-19) in December 2019 in China bestowed a great burden upon children's myopia development and progression. Multiple studies have found myopia prevalence was significantly increased among school-aged children during the home quarantine period (3–8), likely due to behavioral changes induced by home quarantine, including decrease of outdoor activities and increased use of electronic devices (9–20).

The year 2020 is the year of “Quarantine Myopia” (21). Since the outbreak of COVID-19, the virus quickly spread around the world and became a global pandemic. To suppress the virus spreading in the early stage, stringent measures like home quarantine were implemented. During this time, schools in more than 190 countries/territories were partially or fully closed, affecting more than 1.5 billion school-age children (22). In China, students were confined at home taking virtual class from Jan 2020 to Apr 2020. Remote learning, increased use of electronic devices, decreased outdoor time, and lack of organized sports activities were the dominant lifestyle of students during the COVID-19 pandemic (10). In May 2020, schools in China were reopened, and students attended in-person classes the same way as in the pre-COVID period.

In the year 2021, while Delta and Omicron variants deepened the COVID-19 pandemic globally, China had contained the COVID cases to a relatively small number domestically. Schooling and social lives were uninterrupted in general for the whole year of 2021. As a result, China became a natural experiment that provided us a unique opportunity to assess how students' refractive error and axial length progressed at different control phases of the COVID-19 pandemic, and to identify the age at which refractive error was most sensitive to the lifestyle changes that myopia prevention and control should be focusing on.

**Figure 1:**
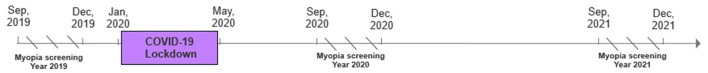
Timeline of COVID-19 lockdown and myopia screenings.

While the impact of lifestyle changes from the COVID-19 pandemic on the incidence and progression of myopia based on non-cycloplegic refractive error (known to over-estimate the myopia prevalence and severity) in school students were reported in several studies (3, 4, 8). the myopic change after the lift of lockdown remains unknown. More importantly, the previous studies have not evaluated the effect of the COVID-19 pandemic on the axial length, an objective measurement that can be accurately measured in children, and is not affected by the cycloplegia (23). We are interested in investigating whether the effect of COVID-19 lockdown on students' myopia persisted as students returned to school, and whether myopia change was accompanied by the increase in axial length. Herein, we conducted a population-based prospective cross-sectional study with three myopia screenings across three years covering before (2019), during (2020), and after (2021) the COVID-19 lockdown as demonstrated in Figure 1. This study aims to evaluate the impact of the COVID-19 lockdown on myopia and axial length of school children in China by comparing them before, during, and after the lockdown.

## Methods

This study is a prospective cross-sectional study started in the Fall of 2019 under the Chinese government's initiative on “National Screening and Intervention of Common Diseases and Health Risk Factors in Students 2019.” The study included three school-based myopia screenings conducted in the Fall of 2019, 2020, and 2021 respectively. Students between the ages of 6 and 12 years old at the date of screening were invited to participate. All data collection and eye examinations followed the tenets of the Declaration of Helsinki. The nature of the study was explained to the participating children and their parents through the school, and verbal informed consent was obtained from parents before the commencement of the study.

### Study subjects

Students from all classes and grades of 88 schools in Qingyang District, Chengdu were invited to participate. Chengdu is the capital city of Sichuan Province in Southwestern China. It is one of the most important economic, financial, commercial, cultural, transportation, and communication cities in China. Chengdu consists of 12 municipal districts with a total population of approximately 20.9 million. Qingyang District is one of 12 districts in Chengdu, located in downtown Chengdu with a population of about 0.96 million. The residents at Qingyang District are mostly of Han ethnicity with a wide spectrum of socioeconomic status. Thus, participants in the study are representative of students in the Southwestern metropolitan area of China. The exclusion criteria were the following: (1) history of ocular surgeries (2) ocular diseases, such as strabismus, amblyopia et al. (3) wore Orthokeratology lenses 4) incomplete measurements or information. Detailed flow chart was shown Supplementary Figure S1.

### Myopia screenings

Three population-based cross-sectional myopia screenings were performed in the Fall (from September to December) of 2019, 2020 and 2021, representing before, during, and after the COVID-19 lockdown in China. A screening team consisting of one certified ophthalmologist, two certified optometrists, and seven certified nurses performed the myopia screening in both eyes, including non-cycloplegic refractive error using NIDEK auto-refractor (ARK-510A; NIDEK Corp., Tokyo, Japan) and axial length using AL-Scan (NIDEK Corp., Tokyo, Japan) following the standard study protocol. As part of the standard screening procedure, the certified ophthalmologist performed slit lamp examination and referred students of any suspicious ocular diseases to eye specialists on-site.

### Statistical analysis

Non-cycloplegic spherical equivalent (SER) in diopters (D) was calculated as sphere plus half of cylinder. Myopia is defined as non-cycloplegic SER ≤ -0.50 D. Continuous measures (e.g., SER, axial length) were summarized by mean ± standard deviation (SD), categorical measures were summarized by count and percentage. For SER and axial length, the average from two eyes was used due to the high inter-eye correlation in SER and axial length (Pearson correlation coefficient = 0.85 and 0.95 respectively).

As age is strongly associated with myopia development and progression (24), we performed age-specific comparison in three different cohorts from 2019 (before COVID-19 lockdown), 2020 (during COVID-19 lockdown) and 2021 (after COVID-19 lockdown). For example, the 6-year-old students in 2019 were compared to 6-year-old students in 2020 (who were 5-year-old in 2019), and compared to 6-year-old students in 2021 (who were 4-year-old in 2019, 5-year-old in 2020). To evaluate the effect of COVID-19 lockdown for each age group of students, we compared the mean SER and mean axial length across three years using analysis of variance (ANOVA), and using chi-square test for comparison of myopia prevalence rate across 3 years (e.g., before, during, and after COVID-19 lockdown). If the overall difference across 3 years is statistically significant (*p* < 0.05), post-hoc pairwise comparisons were made for each pair of years. To assess the overall effect of COVID-19 lockdown on the refractive error and axial length, we performed Poisson regression models for myopia prevalence and generalized linear models for refractive error and axial length. These analyses were adjusted for age and gender, and generalized estimating equations were applied to account for the repeated measures correlation for subjects in more than one myopia screenings. All analyses were conducted using SAS 9.4 (SAS Inst., Cary, NC), and two-sided *p* < 0.05 was considered statistically significant.

## Results

A total of 83,132 students aged 6 to 12 years old were included in the analysis, including 52,748 students in year 2019, 59,002 in year 2020, and 64,368 in year 2021. The mean (SD) age of the students at myopia screening was 8.5 (1.9) years and 52.1% (95% CI: 51.8–52.3%) of the students were male. The distributions of age and gender for each year are shown in Supplementary Table S1.

The myopia prevalence rate was 45.0% (95% CI: 44.6–45.5%) in 2019, 48.7% (95% CI: 48.3–49.1%) in 2020, and 47.5% (95% CI: 47.1–47.9%) in 2021 (Table 1). The overall myopia prevalence rate was significantly higher in 2020 than 2019, and remained higher in 2021 than 2019 (all *p* < 0.001 for comparison with 2019). In analyses stratified by age of students, the greatest increase in myopia prevalence during the COVID-19 lockdown was observed in students 7 years of age, with myopia prevalence of 28.2% in 2019, increased to 33.8% in 2020, and decreased to 31.2% in 2021(all *p* < 0.001 for comparison with 2019). Increase in myopia prevalence after the lift of the COVID-19 lockdown compared to during lockdown was observed in 6-year-old students (from 19.1 to 20.6%, *p* = 0.005). Decrease in myopia prevalence after the lift of the COVID-19 lockdown compared to during lockdown was observed in 9-year-old students (from 55.8 to 54.2%, *p* = 0.03) and 12-year-old students (from 81.0 to 77.7%, *p* < 0.001). Histogram of myopia prevalence across 3 years stratified by age are shown in Supplementary Figure S2.

**Table 1:** Comparisons of myopia prevalence rate across 3 years overall and stratified by age at myopia screening.

	**Myopia (%)**			***p*** **-value** ^*^			
**Age at myopia screening**	**Year 2019**	**Year 2020**	**Year 2021**	**Overall**	**2020 vs. 2019**	**2021 vs. 2020**	**2021 vs. 2019**
6	1,873 (18.7%)	2,036 (19.1%)	2,531 (20.6%)	< 0.001	0.44	0.005	< 0.001
7	2,916 (28.2%)	3,482 (33.8%)	3,435 (31.2%)	< 0.001	< 0.001	< 0.001	< 0.001
8	3,640 (41.6%)	4,589 (44.4%)	4,643 (45.1%)	< 0.001	< 0.001	0.35	< 0.001
9	4,077 (52.9%)	4,738 (55.8%)	5,489 (54.2%)	< 0.001	< 0.001	0.03	0.07
10	4,310 (63.3%)	4,872 (65.8%)	5,357 (64.3%)	0.007	0.002	0.051	0.20
11	3,141 (73.0%)	4,768 (72.9%)	5,162 (71.9%)	0.30			
12	3,792 (79.2%)	4,263 (81.0%)	3,983 (77.7%)	< 0.001	0.03	< 0.001	0.06
All	23,749 (45.0%)	28,748 (48.7%)	30,600 (47.5%)	< 0.001	< 0.001	< 0.001	< 0.001

* From Chi-square test. When overall p-value across 3 years is significant, pairwise comparisons between years were made.

The overall mean (SD) of SER was −0.70 (1.39) D in 2019, and became more myopic with mean (SD) of −0.78 (1.44) D in 2020, and −0.78 (1.47) D in 2021 (all *p* < 0.001 for comparison with 2019) (Table 2). As age increased, the difference in SER across the 3 years decreased, and was not significant in 11 and 12-year-old students. The distributions of SER across 3 years stratified by age are shown in Supplementary Figure S3.

**Table 2:** Comparison of spherical equivalent across 3 years overall and stratified by age at myopia screening.

	**Mean (SD)**			***P*** **–value** ^*^			
**Age at myopia screening**	**Year 2019**	**Year 2020**	**Year 2021**	**Overall**	**2020 vs. 2019**	**2021 vs. 2020**	**2021 vs. 2019**
6	0.01 (0.82)	0.02 (0.87)	−0.02 (0.96)	< 0.001	0.40	< 0.001	0.002
7	−0.19 (0.94)	−0.30 (1.00)	−0.26 (1.03)	< 0.001	< 0.001	0.005	< 0.001
8	−0.49 (1.11)	−0.56 (1.14)	−0.60 (1.18)	< 0.001	< 0.001	0.03	< 0.001
9	−0.81 (1.28)	−0.86 (1.30)	−0.88 (1.35)	< 0.001	0.005	0.44	< 0.001
10	−1.19 (1.48)	−1.24 (1.50)	−1.25 (1.52)	0.03	0.04	0.77	0.02
11	−1.59 (1.67)	−1.58 (1.66)	−1.61 (1.70)	0.57			
12	−1.97 (1.78)	−2.01 (1.83)	−1.98 (1.89)	0.41			
							
All	−0.70 (1.39)	−0.78 (1.44)	−0.78 (1.47)	< 0.001	< 0.001	0.93	< 0.001

* From analysis of variance. When overall p-value across 3 years is significant, pairwise comparisons between years were made.

The overall mean (SD) of axial length was 23.41 (1.01) mm in 2019, 23.45 (1.03) mm in 2020 and 23.46 (1.03) mm in 2021 as shown in Table 3 (all *p* < 0.001 for comparison with 2019). The change of axial length across 3 years followed a similar trend as myopia prevalence and SER. Among the 7-year-old students, mean axial length increased from 23.01 mm in 2019 to 23.05 mm in 2020 (*p* < 0.001), and slightly decreased to 23.04 mm in 2021 (*p* = 0.006 for comparison with 2019). Among 8-year-old students, mean axial length was 23.34 mm in 2019, 23.33 mm in 2020 (*p* = 0.60) and increased to 23.39 mm in 2021 (*p* < 0.001 for comparison to 2019). The distributions of axial length across 3 years stratified by age are shown in Supplementary Figure S4.

**Table 3:** Comparison of axial length across 3 years overall and stratified by age at myopia screening.

	**Mean (SD)**			***P*** **-value** ^*^			
**Age at myopia screening**	**Year 2019**	**Year 2020**	**Year 2021**	**Overall**	**2020 vs. 2019**	**2021 vs. 2020**	**2021 vs. 2019**
6	22.71 (0.72)	22.67 (0.72)	22.70 (0.73)	0.002	< 0.001	0.02	0.19
7	23.01 (0.77)	23.05 (0.78)	23.04 (0.78)	< 0.001	< 0.001	0.17	0.006
8	23.34 (0.85)	23.33 (0.84)	23.39 (0.84)	< 0.001	0.60	< 0.001	< 0.001
9	23.61 (0.92)	23.62 (0.91)	23.64 (0.90)	0.02	0.19	0.12	0.004
10	23.88 (0.96)	23.89 (0.97)	23.92 (0.97)	0.02	0.89	0.02	0.02
11	24.09 (1.01)	24.11 (1.00)	24.14 (1.03)	0.03	0.51	0.051	0.02
12	24.26 (1.07)	24.32 (1.07)	24.31 (1.05)	0.01	0.004	0.56	0.02
							
All	23.41 (1.01)	23.45 (1.03)	23.46 (1.03)	< 0.001	< 0.001	< 0.001	< 0.001

* From analysis of variance. When overall p-value across 3 years is significant, pairwise comparisons between years were made.

In the multivariable analysis of all students adjusted for age and gender, the risk ratio (RR) of myopia was 1.07 (95% CI: 1.06–1.08) in 2020, and 1.05 (95% CI: 1.04–1.06) in 2021 when compared to 2019 (Table 4). Older age (RR = 1.63 to 4.12 for students aged 7–12 compared to 6-year-old, *p* < 0.001), and female gender (RR = 1.10, *p* < 0.001) were significantly associated higher prevalence of myopia. The mean SER was 0.05 D (95% CI: 0.04 D to 0.06 D) more myopic in 2020, and 0.06 D (95% CI: 0.05 D to 0.07 D) more myopic in 2021 when compared to 2019. The mean axial length increased by 0.01 mm (95% CI: 0.01 mm to 0.02 mm) in 2020 and increased by 0.03 mm (95% CI: 0.02 to 0.04 mm) in 2021 when compared to 2019.

**Table 4:** Multivariable analysis for effect of COVID−19 lockdown on myopia, spherical equivalent and axial length adjusted by age and gender.

	**Myopia**		**Spherical equivalent**		**Axial length**	
**Factors**	**Adjusted relative risk (95%CI)**	***P*-value**	**Adjusted Spherical equivalent (95%CI) in diopters**	***P*-value**	**Adjusted Axial length (95%CI) in mm**	***P*-value**
Age at myopia screening		<0.001		<0.001		<0.001
6	Ref		Ref		Ref	
7	1.63 (1.59, 1.67)		−0.26 (−0.27, −0.25)		0.34 (0.33, 0.35)	
8	2.31 (2.26, 2.37)		−0.56 (−0.57, −0.54)		0.66 (0.65, 0.67)	
9	2.90 (2.83, 2.97)		−0.86 (−0.88, −0.84)		0.93 (0.92, 0.94)	
10	3.43 (3.35, 3.51)		−1.23 (−1.26, −1.21)		1.20 (1.19, 1.21)	
11	3.82 (3.73, 3.91)		−1.59 (−1.62, −1.57)		1.42 (1.40, 1.43)	
12	4.12 (4.03, 4.22)		−1.99 (−2.02, −1.96)		1.60 (1.58, 1.62)	
Gender		<0.001		<0.001		<0.001
Male	Ref		Ref		Ref	
Female	1.10 (1.09, 1.11)		−0.09 (−0.11, −0.08)		−0.52 (−0.53, −0.50)	
Time		<0.001		<0.001		<0.001
Year 2019	Ref		Ref		Ref	
Year 2020	1.07 (1.06, 1.08)		−0.05 (−0.06, −0.04)		0.01 (0.00, 0.01)	
Year 2021	1.05 (1.04, 1.06)		−0.06 (−0.07, −0.05)		0.03 (0.02, 0.04)	

## Discussion

In this large prospective population-based cross-sectional study of 83,132 Chinese school students aged 6 to 12 years old, we evaluated the impact of COVID-19 lockdown on development of refractive error and axial length. We found that the COVID-19 lockdown is associated with higher myopia prevalence and longer axial length, and these effects remained 1 year after the lift of lockdown. Significant myopic shift among students during the COVID-19 lockdown was observed, which was consistent with previous studies (3–8). The comparison of results from different studies related to impact of COVID-19 lockdown on myopia is shown in Supplementary Table S2. In our study, the myopia prevalence rate increased by 3.7%, and SER became more myopic by 0.08 D. Unique in our study, we evaluated the impact of COVID-19 lockdown on axial length and found that mean axial length increased by 0.04 mm, supporting the increase in myopic refractive error. Another highlight of our study is that we evaluated the change of refractive error and axial length 1 year after the lockdown was lifted, and found that the impact of the COVID-19 lockdown remained with SER staying the same, and axial length increasing by an additional 0.01 mm in 2021 compared to 2020, despite no home quarantine taking place in Chengdu in 2021.

During the outbreak of COVID-19, many environmental changes occurred including the decrease in outdoor activities and increase in screen time, which may contribute to the increase of myopia shift and elongation of axial length during the home quarantine. Both increasing screen time and reduced time outdoors are strong risk factors for the development of myopia (24). Xu et al. reported Chinese students screen time increased 2.07–3.14 times and outdoor activities time decreased by 1.14–1.71 times during COVID-19 lockdown based on a large-scale survey using questionnaire (8).

After the COVID-19 lockdown was lifted and students returned to in-person school, we expected students to be less myopic compared to during the lockdown; however, we observed the refractive error and axial length remained progressing in 2021 when there was no COVID-19 lockdown in the whole year in China. What are the reasons for the myopic shift after the lift of lockdown? Certain myopia-prone behaviors may persist after the lift of lockdown, but they may not seem be the main reason for the persistent effect. In a recent study from Israel, Shneor et al. showed once students returned to in-person school, the time spent outdoor and amount of physical activities returned to pre-pandemic levels using objective behavior measurements (15). As students returned to school, near work activities were no more than that during the lockdown. Rather, the persistent effect may be the consequence of myopic shift made during the lockdown. Hu et al. studied longitudinal myopia development of students from grade 2 to grade 3 during the COVID-19 lockdown, and found among students who were not myopic before lockdown, the proportion of students with SER between −0.50 to +0.50 D increased from 30 to 50% comparing to 2019 (7). Because students with SER below “age-normal hyperopia” (i.e., below +0.50 D for ages 7 to 8 years, below +0.25D at ages 9 to 10 years and emmetropia at age 11 years) were at significant risk of developing myopia (25), the COVID-19 lockdown might have substantially increased the proportion of students that had SER drop below such borderline. Therefore, both myopia and axial length could still increase even after the lift of COVID-19 lockdown, when students returned to in-person school.

In our study, the effect of COVID-19 lockdown on myopia was observed in all age groups, but not to the same magnitude. The 7-year-old students showed the greatest changes of myopia from the COVID-19 lockdown, with myopia prevalence rate of 28.2, 33.8, and 31.2% respectively before, during and after lockdown. Students in other age groups were less affected. Wang et al. similarly reported that home quarantine has most substantial impact on the myopia risk for students of age 6–8 years old (3). However, the magnitude of myopic shift during COVID-19 lockdown in our study was much smaller comparing to Wang et al.'s results (13.6 in 2019 and 26.2% in 2020 for 7-year-old students) (3). We speculate the reasons may be the following: 1) the time of myopia screening in 2020 took place during September to December 2020 in our study, whereas the photoscreening in Wang et al.'s study was in June 2020 (3). In Chang et al.'s study, the author found a “hyperopic progression” from May to October 2020 which may be explained by short-term accommodative spasm (4). The myopia screening immediately after the COVID-19 lockdown may overestimate the actual myopic shift among students. (2) The difference may be due to myopia screening methods. Our study used NIDEK autorefractor, while Wang et al. used Photoscreening. These devices have different sensitivity and specificity for detecting myopia. (3) The myopia prevalence rate before the COVID-19 outbreak was much higher in our study than the rate in Wang et al.'s study. This difference may due to cohort difference. Wang et al. included students from Feicheng, a county-level city in Northern China and our cohort was from Chengdu, a capital city in which students were more likely exposed to electronic devices early in life and less per capita outdoor area compared to Feicheng. Notably a large cohort from Wenzhou city in Xu et al.'s study, the results were closer to our study (overall myopia prevalence among primary school students from 34.4 to 42.8%, comparing to our study from 45.0 to 48.7% in myopia rate before and during COVID-19 lockdown) (8). (4) The smaller degree of myopic shift in our study during COVID-19 lockdown than other studies may partially be explained by well-implemented school-based eye health education in Qingyang district of Chengdu. A 2-year randomized clinical trial by Li et al. showed school-based family health education was effective in myopia prevention and control (26). Since 2019, large amount of work for promoting myopia-control education has been consistently implemented in Qingyang district. In each school semester, seminars on myopia prevention and control were presented by experts to students, their parents and teachers in Qingyang district. Educational videos, comics, live streaming of famous ophthalmologists/optometrists and other educational materials for promoting myopia prevention and control were also used through social medias (eg., WeChat).

Our study showed the effect of COVID-19 lockdown on students' myopic shift was accompanied with elongation of axial length. We were concerned with the high myopia prevalence in these young students, because early onset of myopia was associated with a higher risk of subsequently developing high myopia (27). Given that children typically progress from hyperopia to emmetropia in ages 5–12 (28), reducing near-work time and increasing time on outdoor activities should be encouraged, especially among lower grade students. A study in Taiwan for promoting outdoor activities among preschoolers showed outdoor activities effectively reduced myopia prevalence from 15.5 in 2014 to 10.3% in 2020 (29). Implementing a myopia prevention program among preschoolers, especially in areas with a high prevalence of myopia early at age 6, should be encouraged.

### Limitations

To our knowledge, this study represents one of the largest samples of Chinese students aged 6–12 years with axial length data and provides the first population-based evidence of students' myopia status and progression after the lift of the COVID-19 lockdown. However, this study has limitations. First, our study did not collect any lifestyle data, including near-work and outdoor activities, which limit our interpretation of observed changes in refractive error and axial length. Second, similar to other previous large studies of myopia during the COVID-19 pandemic (3, 4, 8), our refractive error was measured without administration of cyclopegia, which were reported to overestimate myopia prevalence (30). However, this limitation is remedied by the axial length measurement, which was not impacted by the cycloplegia (23). Third, our study was limited to school students aged from 6 to 12 years old, however the age range are most sensitive to the impact from intervention or environmental factors.

## Conclusions

In conclusion, this large population-based cross-sectional study across 3 years of before, during, and after COVID-19 lockdown found the COVID-19 outbreak had significant impact on the development of refractive error and axial length of- Southwestern Chinese school students, and its impact remained even 1 year after the COVID-19 lockdown was lifted. Longitudinal studies for following-up with these students impacted by COVID-19 outbreak will help us further understand its long-term effect on refractive error and vision.

## Data availability statement

The datasets are not available to the public because confidentiality agreement with the Education Bureau. The study is collaborating with the Qingyang Education Bureau under the Chinese government's initiative on “National Screening and Intervention of Common Diseases and Health Risk Factors in Students 2019”. For any potential collaboration, please sent request to the corresponding author Dr. XL (lixiaoning@aierchina.com).

## Ethics statement

The studies involving human participants were reviewed and approved by Committee of Research Ethics of the Aier Eye Hospital Group Ethical Committee, Hunan, China (ID:AIER2019IRB05). The nature of the study was explained to the participating children and their parents through the school, and verbal informed consent was obtained from parents before the commencement of the study.

## Author contributions

ZY, WL, and XL designed the study. WL, JL, and LZ directed the study's implementation. WP and GY designed the analytical strategy and helped to interpret the findings. WP prepared the draft. All authors helped to review the manuscript.
