# Supplementary material for: Myopia and axial length in school-aged children before, during, and after the COVID-19 lockdown–A population-based study
Source: Front Public Health. 2022 Dec 15;10:992784. doi: 10.3389/fpubh.2022.992784 (PMC9799254; doi:10.3389/fpubh.2022.992784)
Supplement: Supplementary file 1 [file Table_1.DOCX]

***eTable 1: Demographics***

| **Patient Characteristics** | **Year 2019** | **Year 2020** | **Year 2021** | **P-value** |
| --- | --- | --- | --- | --- |
| Number of students | 52748 | 59002 | 64368 |  |
|  |  |  |  |  |
| Age (years) |  |  |  |  |
| Mean (SD) | 8.4 (1.9) | 8.5 (1.9) | 8.5 (1.9) | <0.001 |
| 6 | 10030 (19.0%) | 10664 (18.1%) | 12307 (19.1%) |  |
| 7 | 10354 (19.6%) | 10305 (17.5%) | 10993 (17.1%) |  |
| 8 | 8754 (16.6%) | 10334 (17.5%) | 10306 (16.0%) |  |
| 9 | 7713 (14.6%) | 8489 (14.4%) | 10126 (15.7%) |  |
| 10 | 6807 (12.9%) | 7402 (12.5%) | 8327 (12.9%) |  |
| 11 | 4304 (8.2%) | 6543 (11.1%) | 7183 (11.2%) |  |
| 12 | 4786 (9.1%) | 5265 (8.9%) | 5126 (8.0%) |  |
|  |  |  |  |  |
| Gender |  |  |  | 0.23 |
| Male | 27431 (52.0%) | 30803 (52.2%) | 33504 (52.1%) |  |
| Female | 25317 (48.0%) | 28199 (47.8%) | 30864 (47.9%) |  |

***eTable 2: Results of impact of COVID-19 lockdown on students’ myopia from different research articles***

| **Author** | **Settings** | **Prevalence before**  **lockdown** | **Prevalence during lockdown** | **Change in Refractive error** | **Change in Axial length** |
| --- | --- | --- | --- | --- | --- |
| Wang J, et al. (2021)^3^ | Cross-sectional;  123,535 students aged 6-13 years;  results at Sep 2019 compare to results at June 2020; Age-specific comparison; Without Cycloplegia | aged 6 (5.7%), 7 (13.6%), 8 (26.3%), 9 (38.8%), 10 (50.7%), 11 (62.5%), 12 (69.8%), 13 (82.1%) | aged 6 (21.5%), 7 (26.2%), 8 (37.2%), 9 (45.3%), 10 (52.8%), 11 (59.5%), 12 (67.1%), 13 (81.5%) | aged 6 (-0.32D), 7 (-0.28D), 8 (-0.29D), 9 (-0.14D), 10 (-0.14D), 11 (-0.06D), 12 (-0.05D), 13 (-0.05D) | N/A |
| Chang, et al. (2021)^4^ | longitudinal;  29,719 students with mean (SD) age of 9.9 (2.0) years at Sep 2019 compare to same students with mean (SD) age of 10.5 (2.0) years at May 2020; Same-subject comparison; Without Cycloplegia | 53.2% overall | 73.7% overall | -0.50 D overall | N/A |
| Xu, el at. (2021)^8^ | longitudinal;  1,001,749 students aged 7-18 years eligible at baseline; Results at Dec 2019 were compared to results at June 2020; Without Cycloplegia | 34.4% in elementary school students | 42.8% in elementary school students | -0.113 D overall | N/A |
| Hu, et al. (2021)^7^ | Longitudinal; 1,472 grade two students observed from Dec 2018 to Dec 2019 (non-exposure group) were compared to 1,207 grade two students observed from Dec 2019 to Dec 2020 (exposure group); Between-cohort comparison; With Cycloplegia | 13.3% in non-exposure group at Dec 2019 | 20.8% in exposure group at Dec 2020 | -0.35 (-0.45 to -0.25) D | -0.02 (-0.08 to 0.05) |
| Wang W, et al. (2021)^10^ | Cross-sectional;  1,728 students (from primary to high school) filled survey in Oct 2019 were compared to 1,733 students (same sample) filled survey in June 2020; Without Cycloplegia | 28% among primary school students | 39% among primary school students | -0.30 overall | N/A |
| Cai, et al. (2022)^31^ | longitudinal;  115 myopic students with mean±SD age of 9.34±2.00 who had replaced frame-glasses in Dec 2019 (before COVID-19) were examined at Feb 2020 (beginning of lockdown) and May 2020 (lockdown lifted); Same-subject comparison; Without cycloplegia | N/A | N/A | -0.45 D in 3 months | 0.14 mm in three months |
| Ma D, et al. (2021)^13^ | longitudinal;  208 students aged 8 to 10 years examined at July 2019, Jan 2020 and Aug 2020; Same-subject comparison; With cycloplegia | 58.7% | 85.6% | -0.93 D | 0.24 mm |
| Ma M, et al. (2021)^16^ | longitudinal;  201 myopic students aged 7 to 12 years examined at Apr 2019, Oct 2019 and May 2020; Same-subject comparison; With cycloplegia | N/A | N/A | -0.98 D | N/A |
| Zhang, et al. (2021)^6^ | longitudinal;  709 students aged 6 to 8 years examined at Dec 2019, Jul 2020 (Covid-19 cohort) were compare to 1084 students aged 6 to 8 years who were follow since Mar 2018 for three years (pre-Covid-19 cohort); Between-cohort comparison; With cycloplegia | 18.97%  in Covid-19 cohort | 35.25%  in Covid-19 cohort | -0.50 D within the Covid-19 cohort | 0.29 mm within the Covid-19 cohort |
| Alvarez-Peregrina C, et al. (2021)^5^ | Cross-sectional;  4,227 children aged 5-7 years screened in Oct 2019 were compared to 1,600 children aged 5-7 years screened in Oct 2020; Without Cycloplegia | 21% overall | 20% overall | -0.18 D overall | N/A |
| Our study | Cross-sectional;  5,2748 students aged 6-12 years in Fall 2019 (Before lockdown) were compared to 59,002 students aged 6-12 years in Fall 2020 (During lockdown) and 64,368 students aged 6-12 years in Fall 2021 (After lockdown); Age-specific comparison; Without Cycloplegia | 45.0% overall | 48.7% overall | -0.08 D overall | 0.04 mm overall |

***eFigure 1: Flow chart***

***eFigure 2: Histograms of Myopia prevalence across 3 years stratified by age***

***eFigure 3: Distribution of Spherical Equivalent across 3 years stratified by age***

eFigure 3. The vertical line in the distribution represents the mean.

***eFigure 4: Distribution of Axial Length across 3 years stratified by age***

eFigure 4. The vertical line in the distribution represents the mean.
