# Supplementary figures and images for: Myopia and axial length in school-aged children before, during, and after the COVID-19 lockdown–A population-based study
Source: Front Public Health. 2022 Dec 15;10:992784. doi: 10.3389/fpubh.2022.992784 (PMC9799254; doi:10.3389/fpubh.2022.992784)

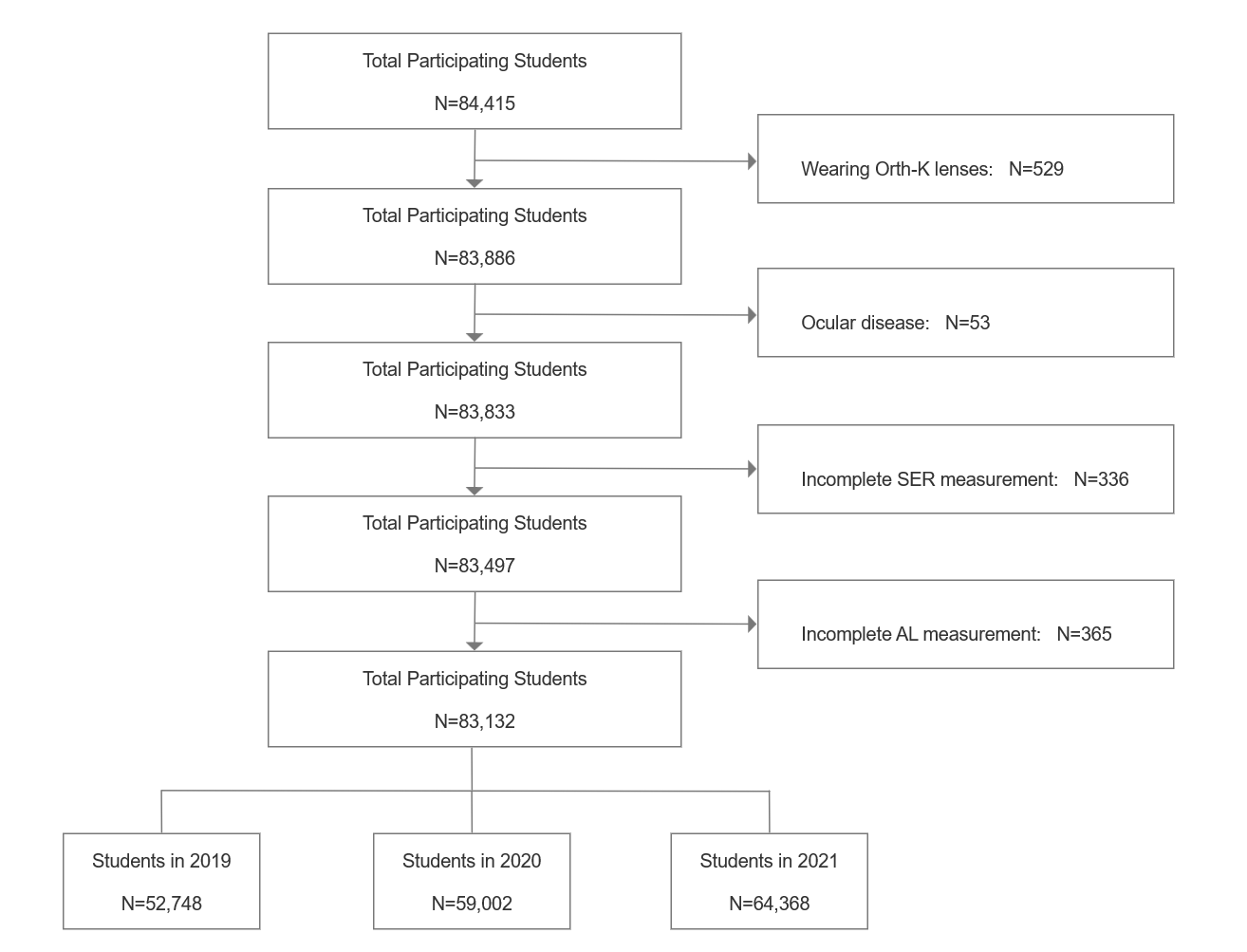

Supplement: Supplementary file 2 [file Presentation_1.ZIP › eFigure1.png]

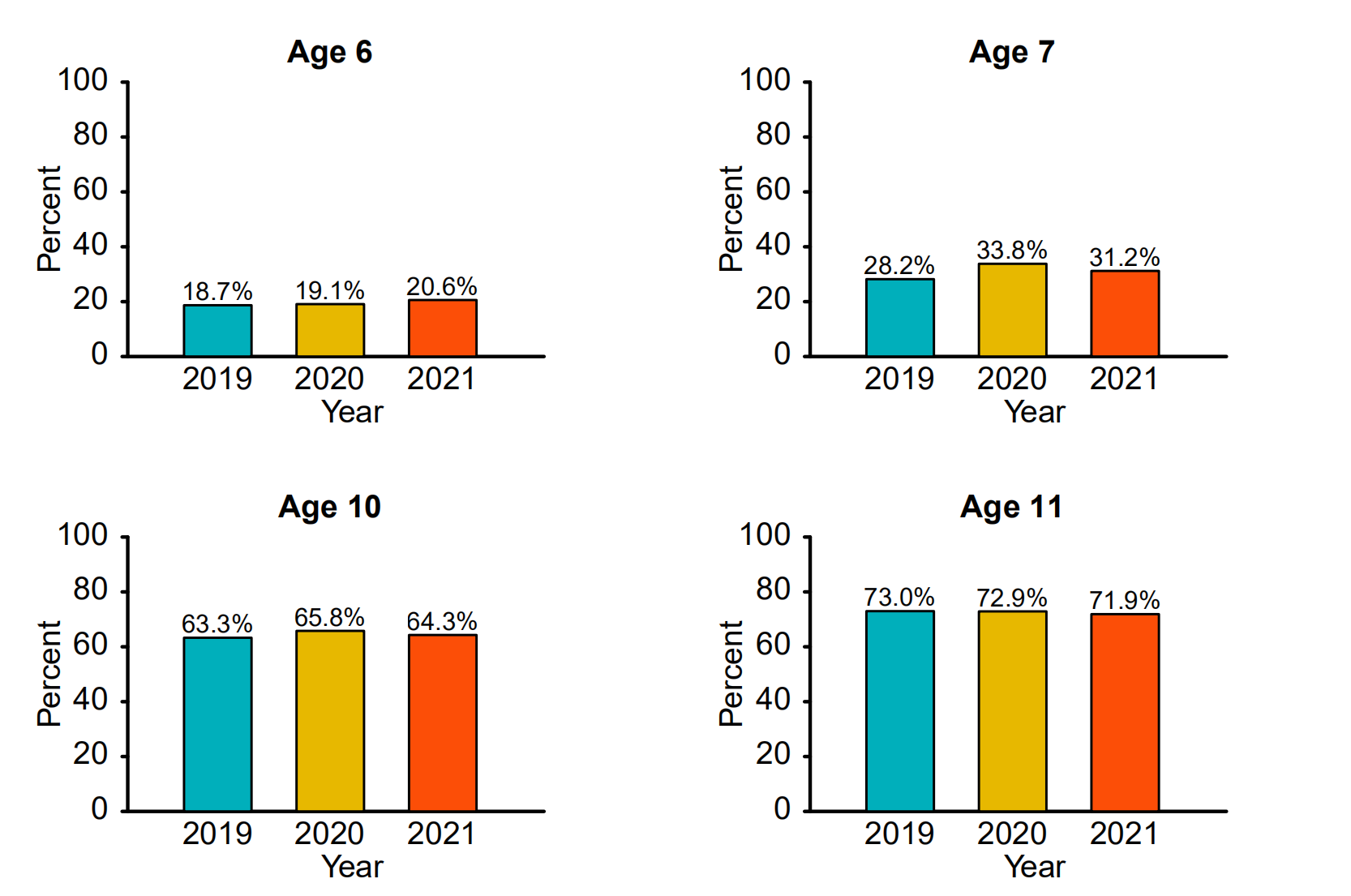

Supplement: Supplementary file 2 [file Presentation_1.ZIP › eFigure2a.png]

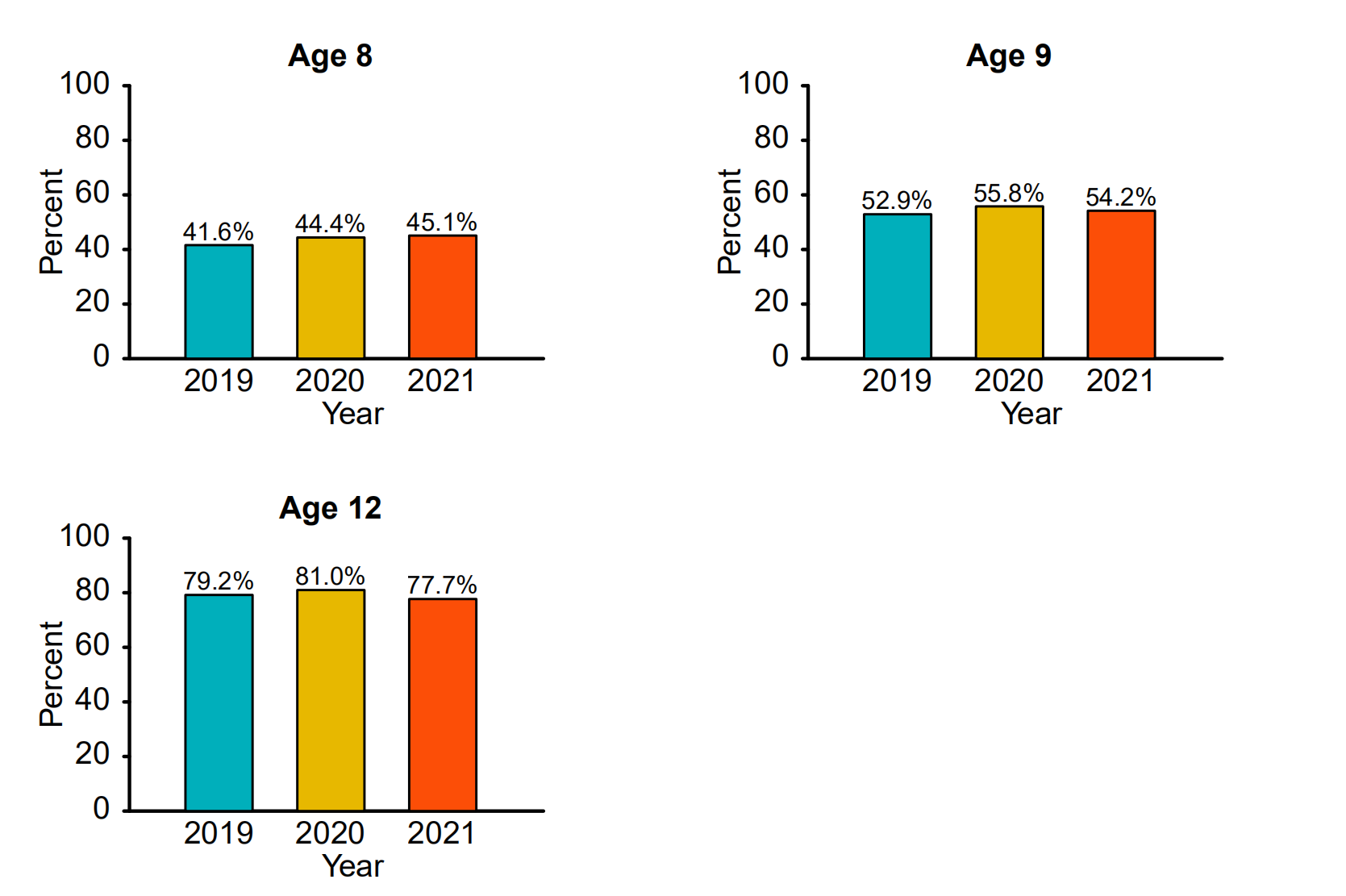

Supplement: Supplementary file 2 [file Presentation_1.ZIP › eFigure2b.png]

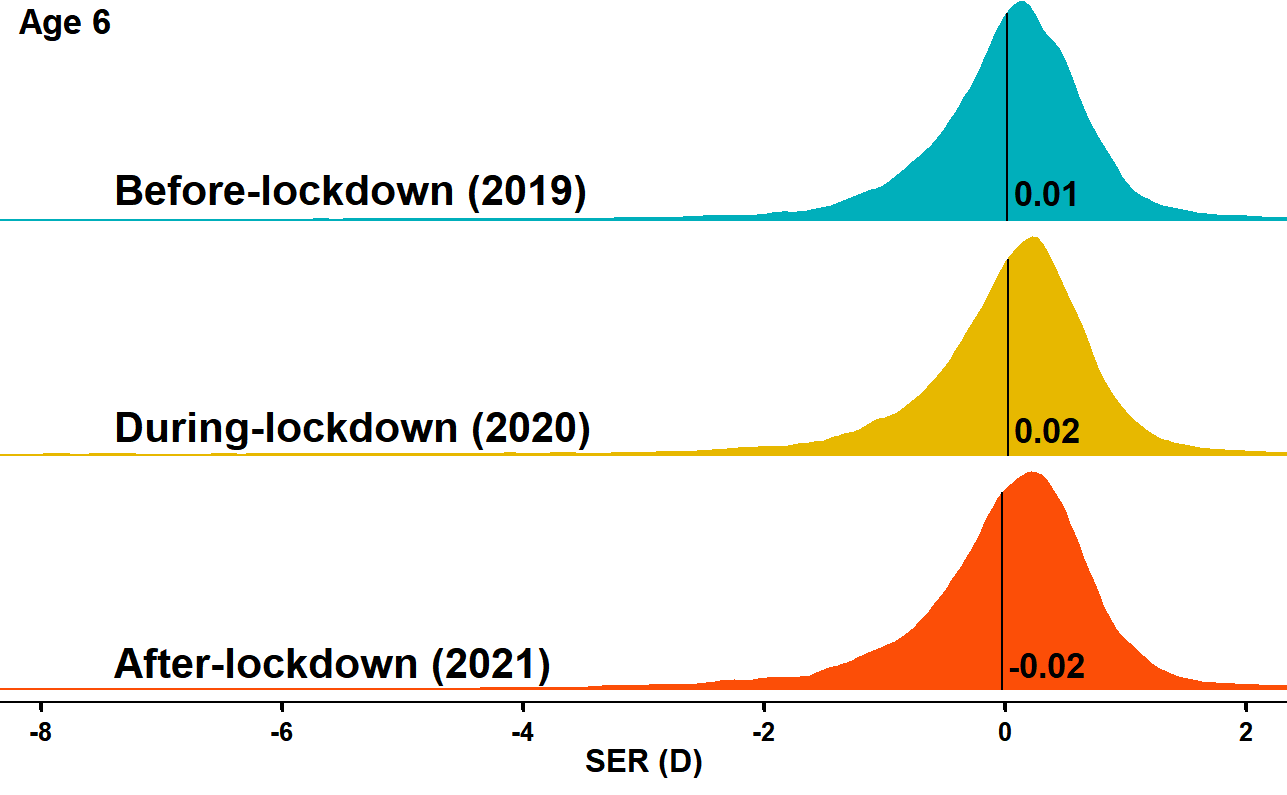

Supplement: Supplementary file 2 [file Presentation_1.ZIP › eFigure3a.png]

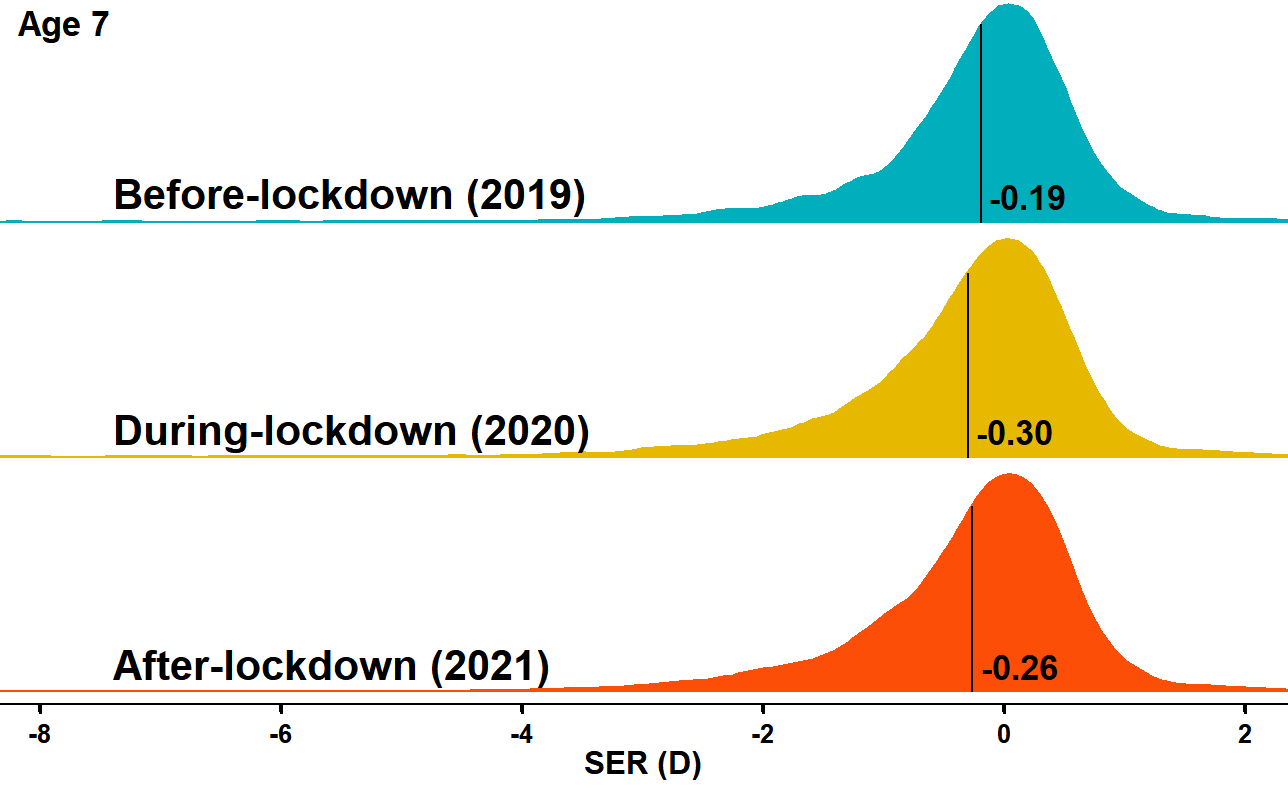

Supplement: Supplementary file 2 [file Presentation_1.ZIP › eFigure3b.png]

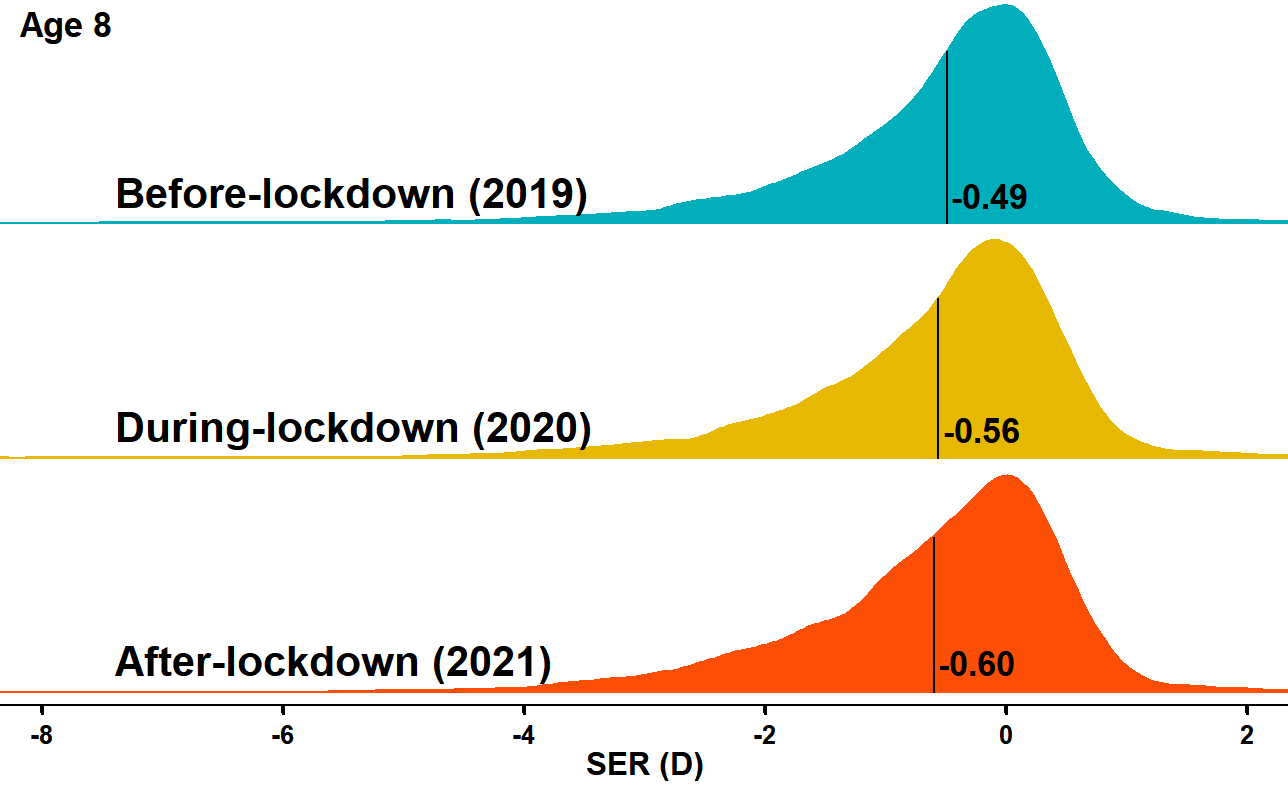

Supplement: Supplementary file 2 [file Presentation_1.ZIP › eFigure3c.png]

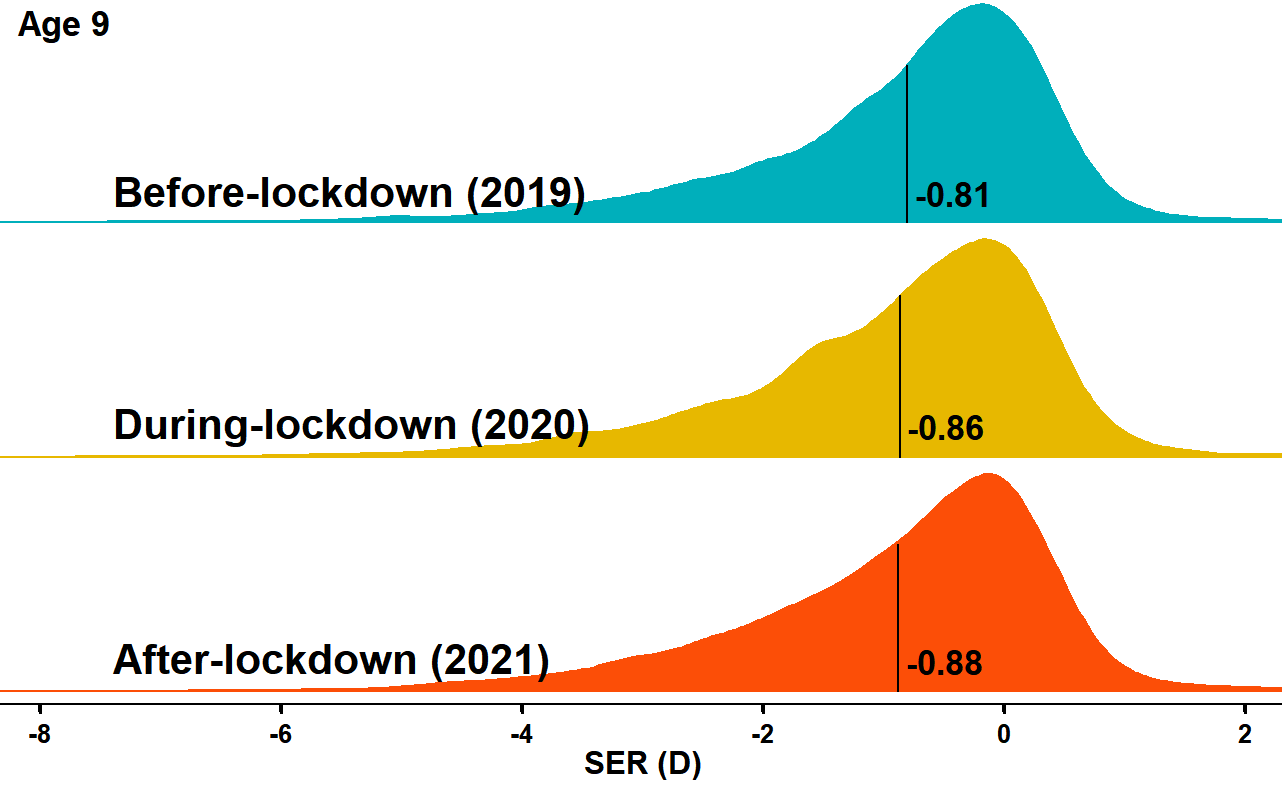

Supplement: Supplementary file 2 [file Presentation_1.ZIP › eFigure3d.png]

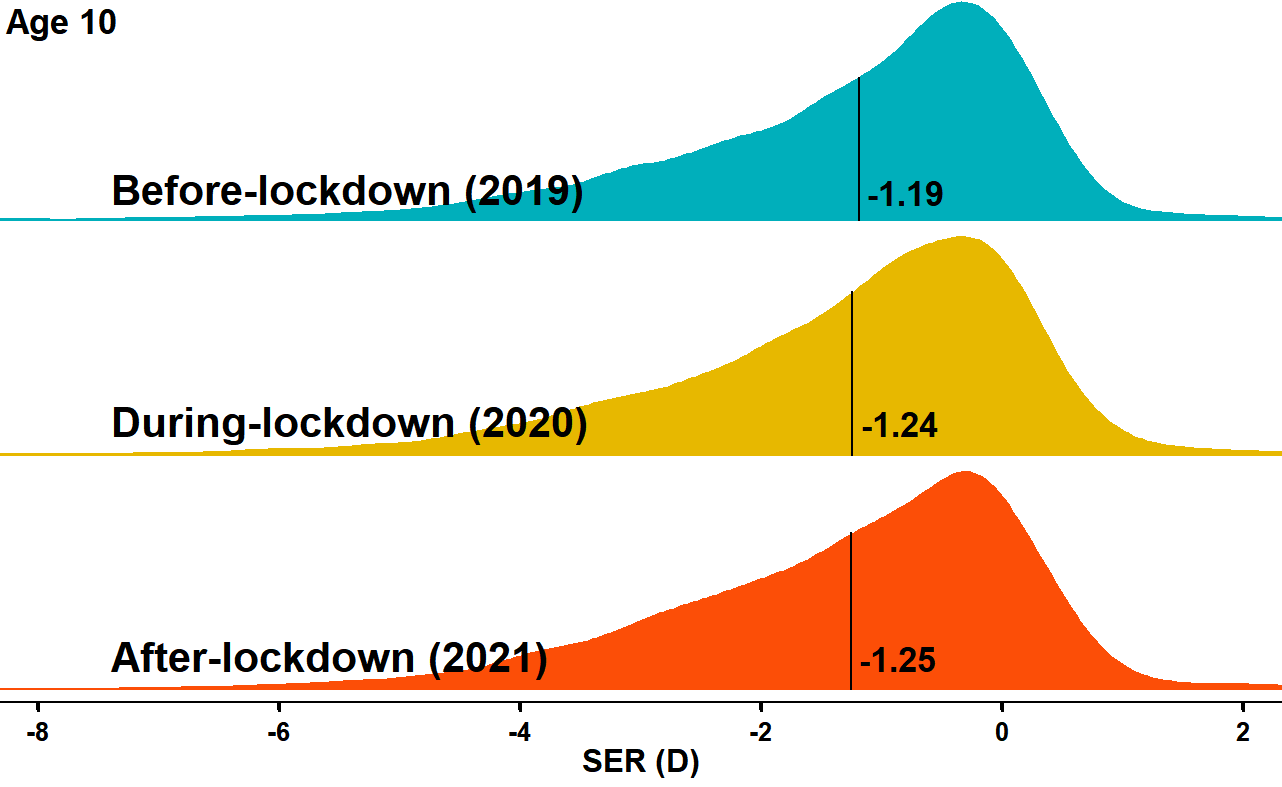

Supplement: Supplementary file 2 [file Presentation_1.ZIP › eFigure3e.png]

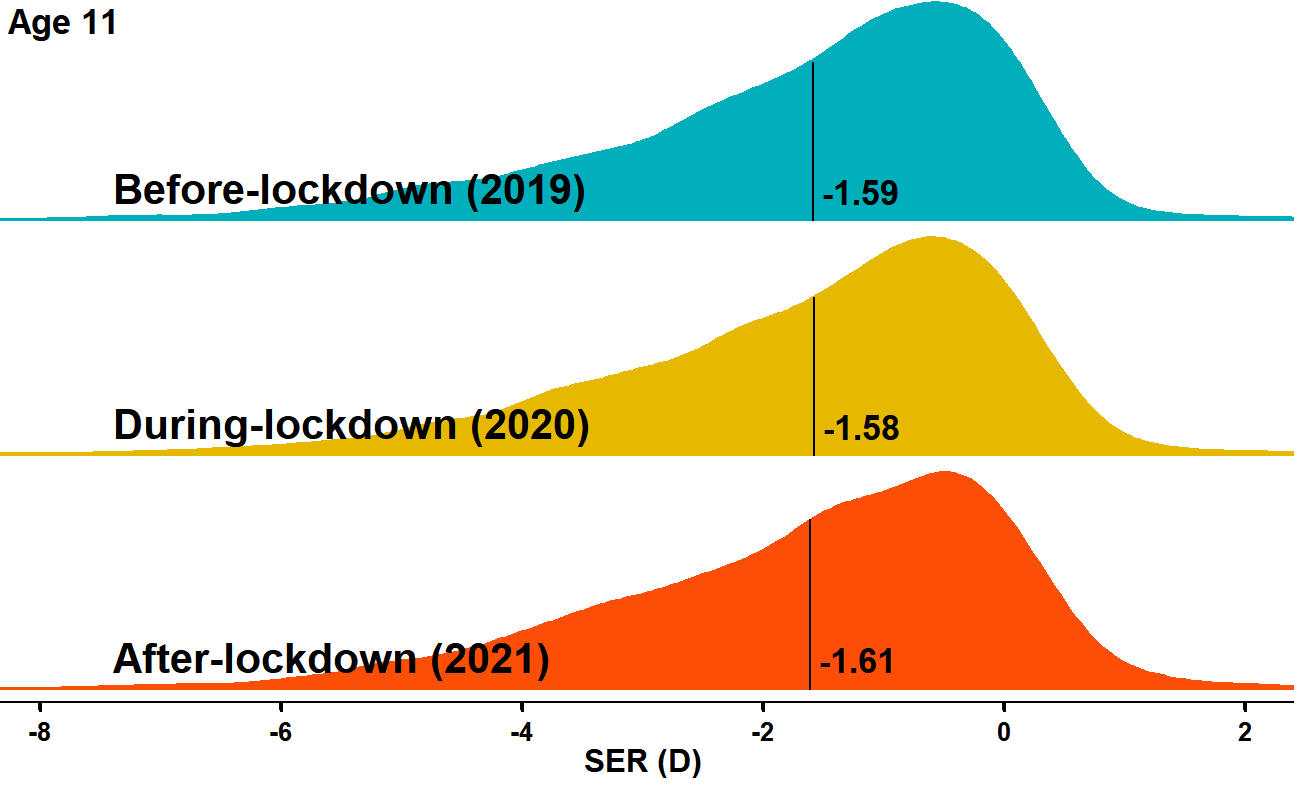

Supplement: Supplementary file 2 [file Presentation_1.ZIP › eFigure3f.png]

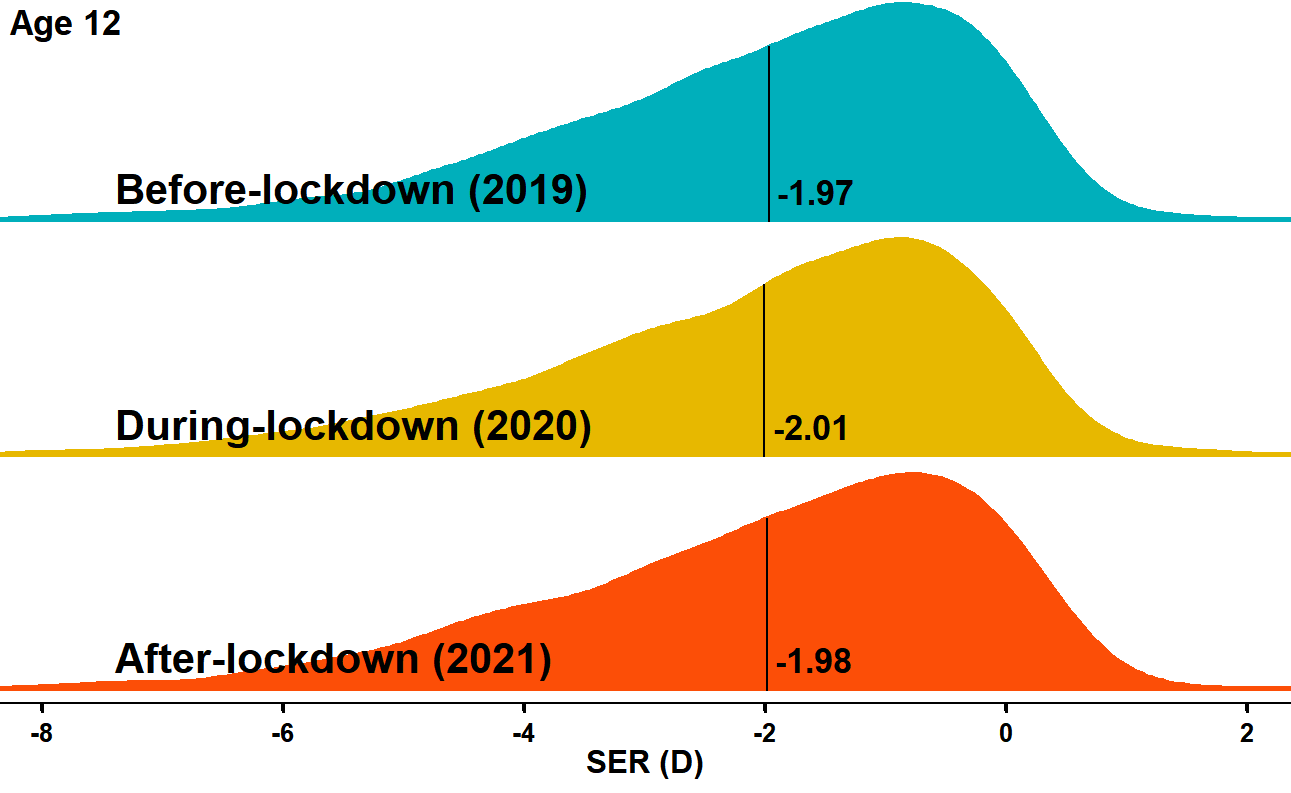

Supplement: Supplementary file 2 [file Presentation_1.ZIP › eFigure3g.png]

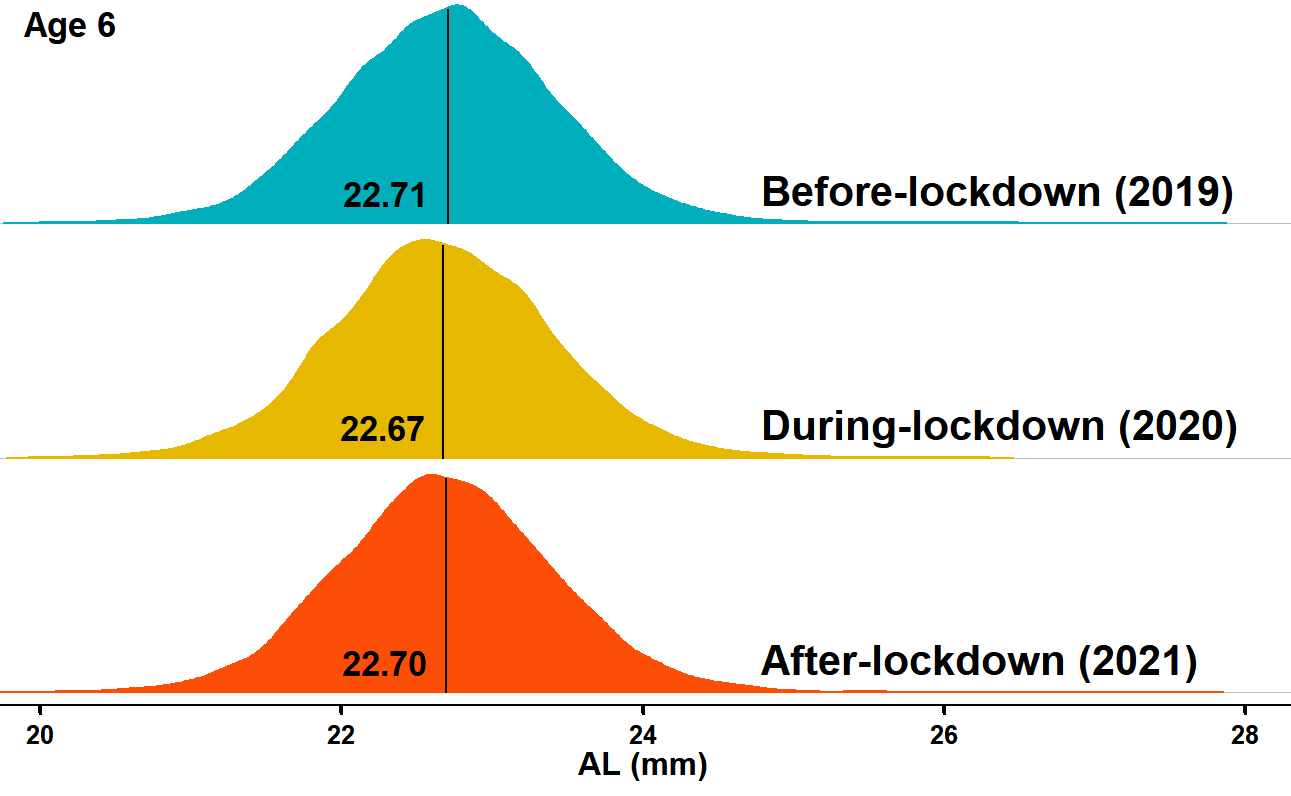

Supplement: Supplementary file 2 [file Presentation_1.ZIP › eFigure4a.png]

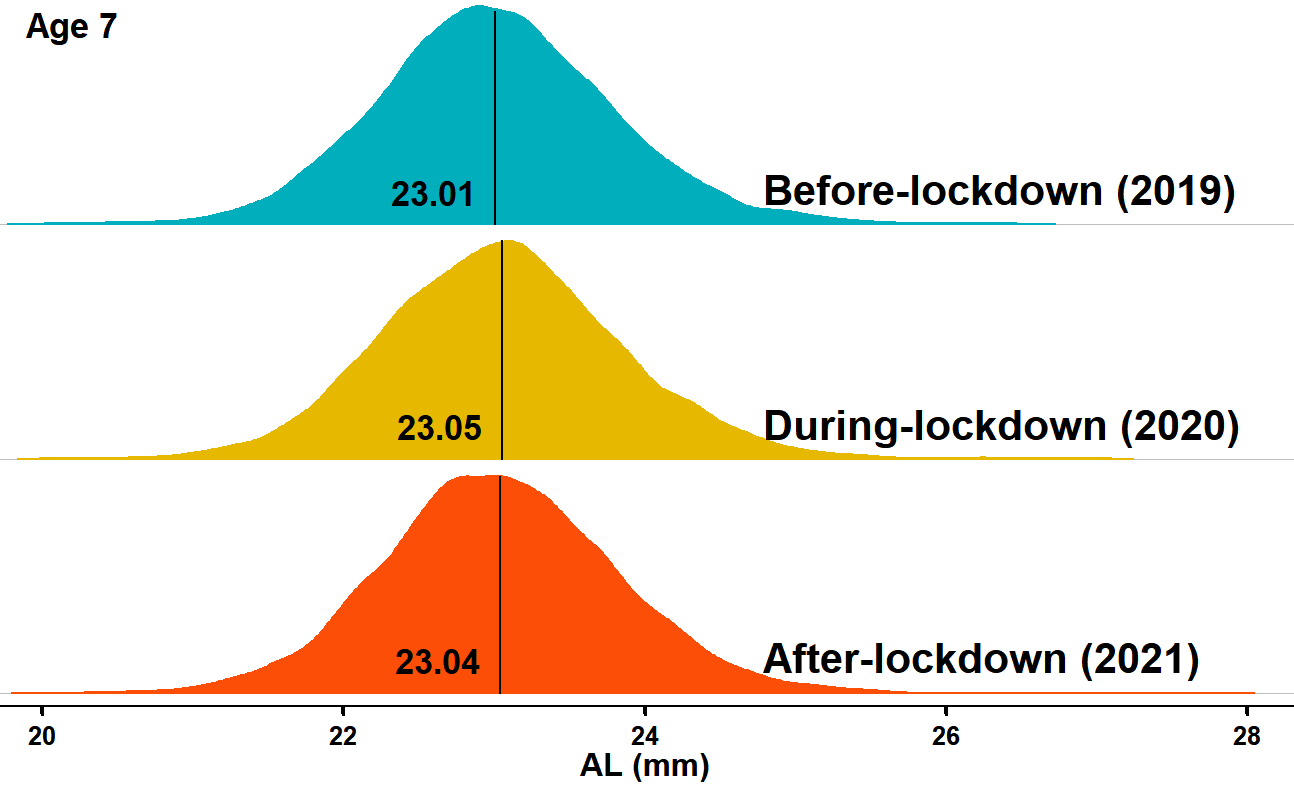

Supplement: Supplementary file 2 [file Presentation_1.ZIP › eFigure4b.png]

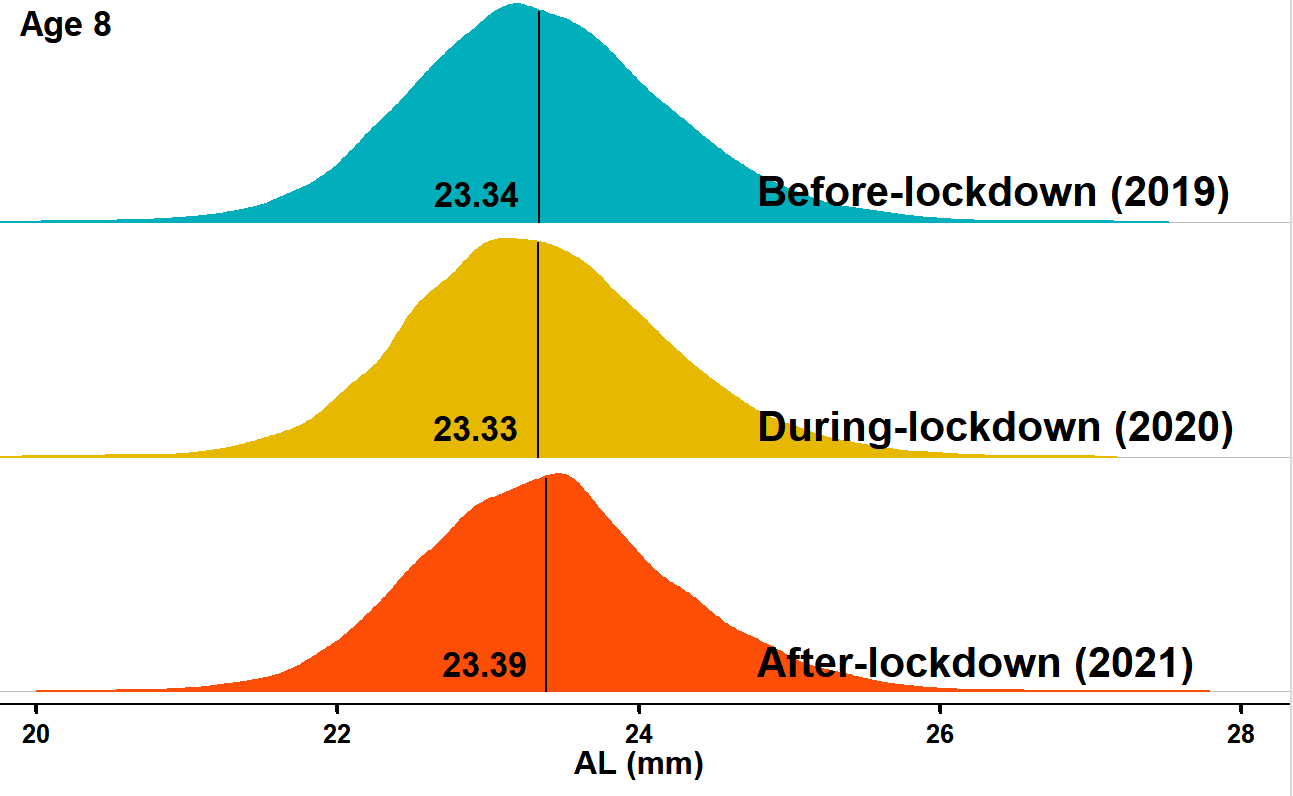

Supplement: Supplementary file 2 [file Presentation_1.ZIP › eFigure4c.png]

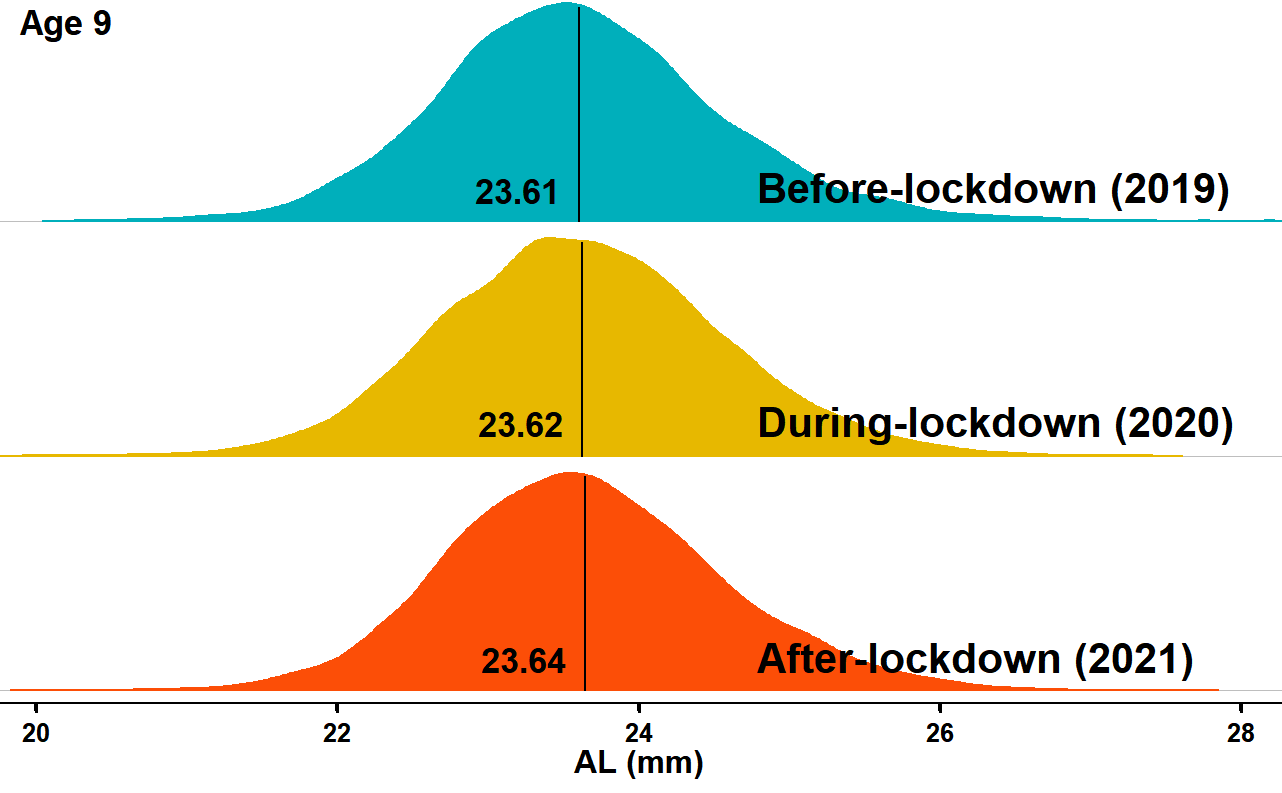

Supplement: Supplementary file 2 [file Presentation_1.ZIP › eFigure4d.png]

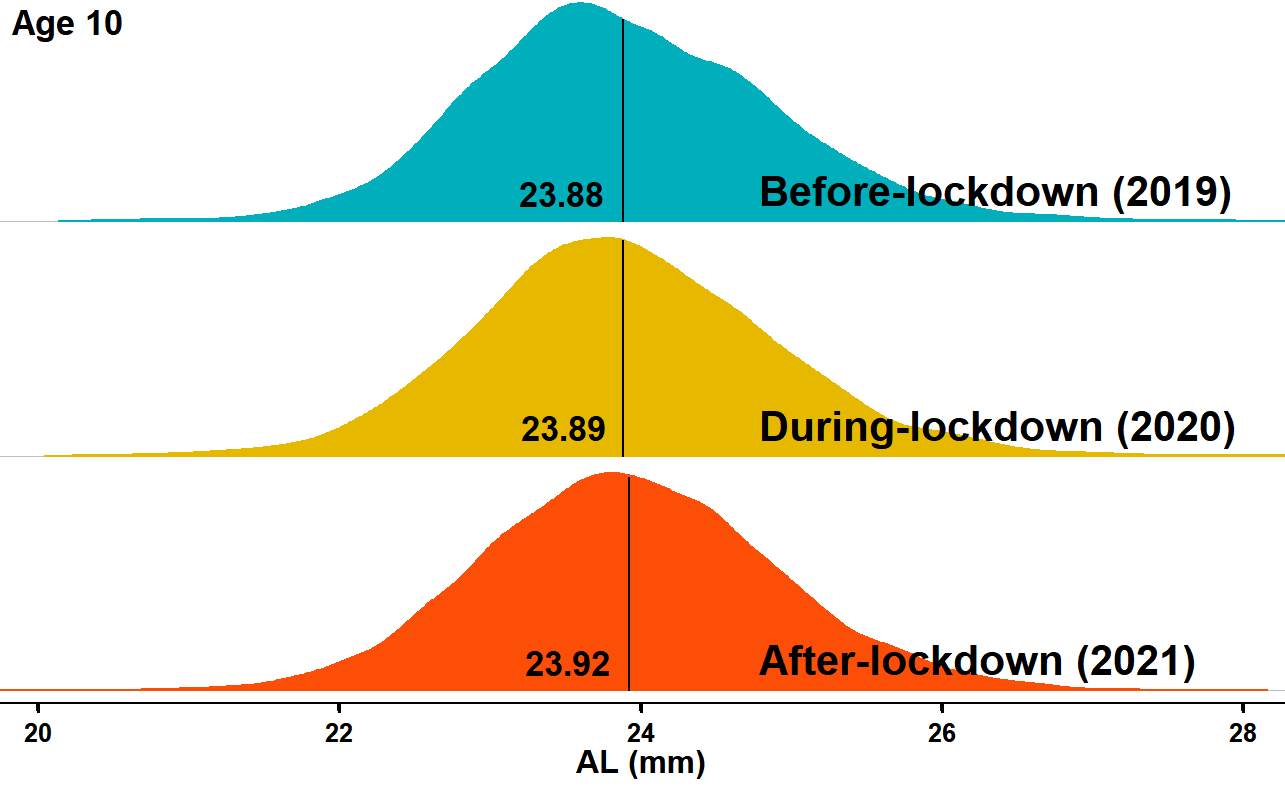

Supplement: Supplementary file 2 [file Presentation_1.ZIP › eFigure4e.png]

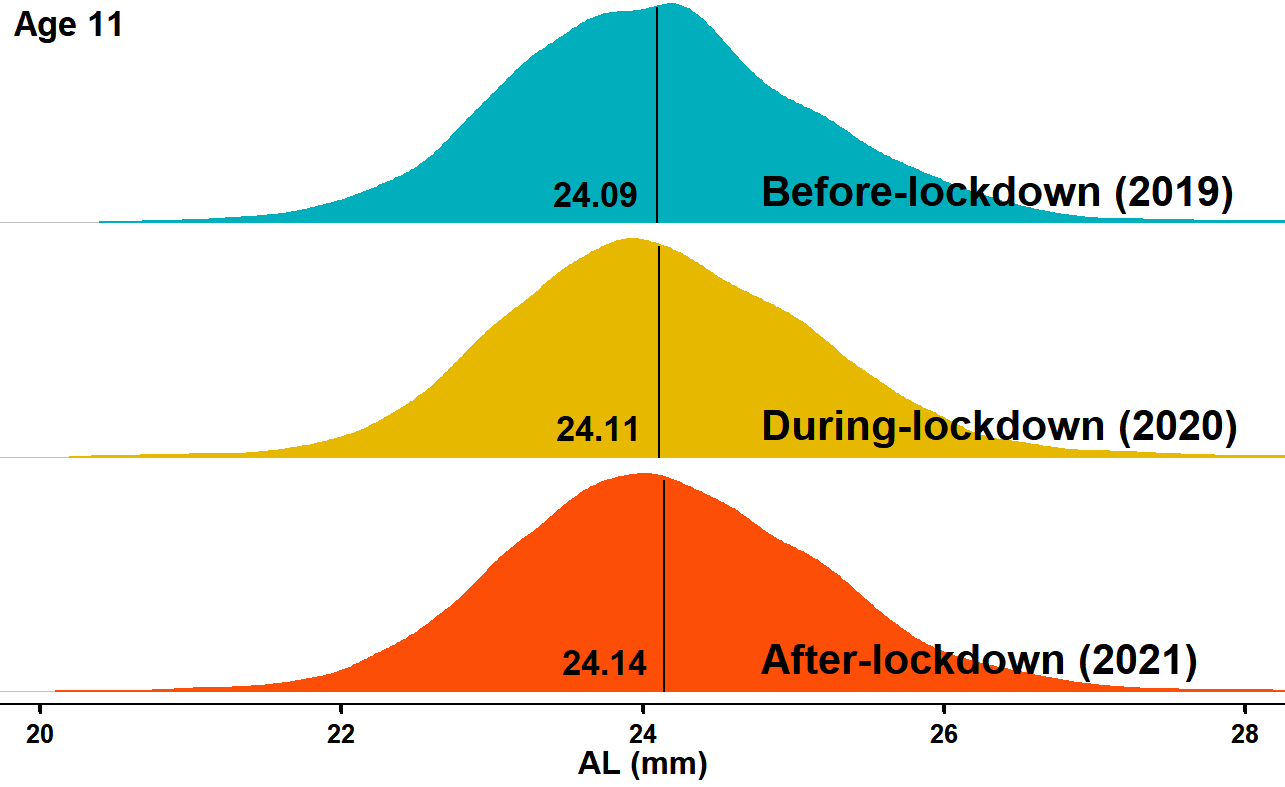

Supplement: Supplementary file 2 [file Presentation_1.ZIP › eFigure4f.png]

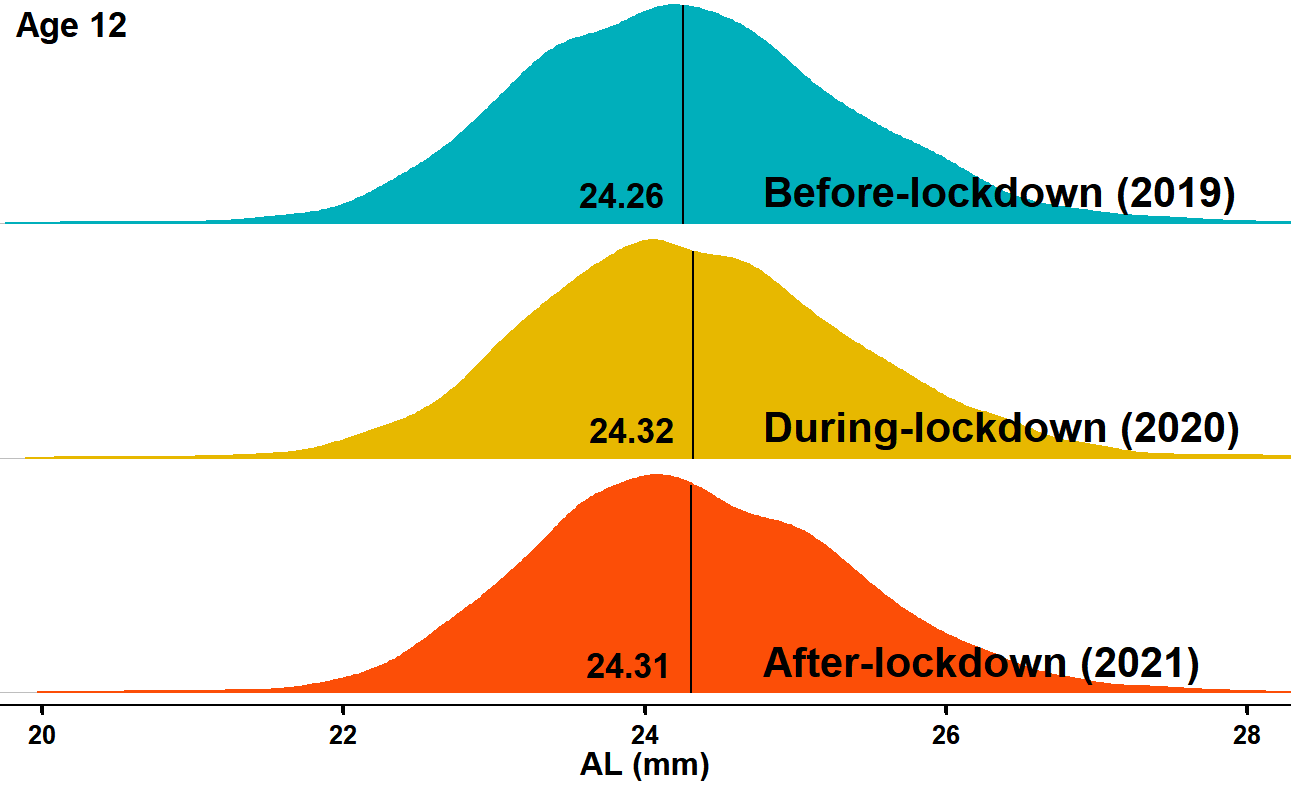

Supplement: Supplementary file 2 [file Presentation_1.ZIP › eFigure4g.png]
